# A pessimistic View on the Future of Drugs: Pharmapolicy Determines Whether New Drugs will Become Available

**DOI:** 10.1155/S0962935194000128

**Published:** 1994

**Authors:** H. Timmerman

**Affiliations:** Leiden/Amsterdam Center for Drug Research Vrije Universiteit Department of Pharmacochemistry De Boelelaan 1083 Amsterdam 1081 HV The Netherlands


Mediators of Inflammation **3**, 107–110 (1994)

### Editorial Message

Our Journal, now in its third year, has earned for itself a reputation of being the fastest forum for disseminating the results of research on inflammation processes, in particular within the field of mediators. This field continues to undergo a spectacular growth in several directions (immunopathology, allergology, joint conditions, pulmonary disorders, transplantation etc.), all of which are well represented in reviews and research articles of *Mediators of Inflammation*. Increased knowledge about mediators has also a considerable impact on finding and developing improved drugs for treating the above conditions. Accordingly, articles dealing with this theme would be particularly welcome in our Journal. To this end we invited Professor. H. Timmerman, a leading expert in pharmacochemistry and drug development, to put forward his personal view on this subject. We hope that this Debate Article will precipitate a response from research workers of the pharmaceutical industry. Responses to this article and/or other submissions around this subject would be encouraged by the Editors.

### A pessimistic view on the future of drugs: pharmapolicy determines whether new drugs will become available

**H. Timmerman**

Leiden/Amsterdam Center for Drug Research, Vrije Universiteit, Department of Pharmacochemistry, De Boelelaan 1083, 1081 HV Amsterdam, The Netherlands

Throughout the existence of mankind, there has been a need for treating—and if possible, for curing—diseases. One of the most explored possibilities is treatment by means of medicinal agents. Traditionally, medicinal agents were found in natural sources, mainly in plants, but, occasionally, such products were obtained from animals. A number of so-called modern therapeutics have a long history and have been developed from traditional medicines; some very well-known examples are acetylsalicylic acid; the heart glycosides, such as digitoxin; and the anticancer drug, etoposide.

Only relatively late in history were medicinal agents selected on the basis of certain properties of the preparation itself or its source. A well-known example is the use of the mandrake; this plant has roots the shape of which has something in common with the shape of the human body; this fact was sufficient to consider it a cure for any kind of ailment.

Increasing insight into the functioning of the human body and the causes of disease have allowed us to develop more effective drugs. One limiting factor has often been the inadequate knowledge of what we now call 'pure' chemical products. Until about 1850 chemistry was dominated by inorganic and analytical chemistry. Chemicals which were applied as drugs were, accordingly, inorganic compounds. Such compounds (especially metal oxides) are generally rather toxic and produce mainly signs of toxicity, making believe that the treatments were effective. The tradition of using these inorganic substances led people such as Paracelsus and Hahnemann to advocate the use of highly diluted preparations, which eventually led to the development of the homeopathic principle. The principle of agents that worked but not helped has in the course of time been transferred into the use of preparations that sometimes helped, but not worked.

Around 1850 synthetic organic chemistry started to develop. At almost the same time experimental pharmacology was introduced, and therefore the hope for new medicines became extremely high at that time. For the first time chemistry played its own role in the development of new drugs.

From 1850 onwards chemists made large numbers of new organic compounds. Pharmacologists established whether these substances, when administered to animals, caused any effects which could be of potential interest for influencing a certain physiological function and which, subsequently, could be regarded as a cure for a certain disease. This procedure (synthesizing and 'screening') was scientifically not very appealing, but did lead to a large number of highly effective medicines, some of which are still in use. In this way the first antihistamines ($H_1$-blockers), the anti-allergic drug cromoglycate and the non-steroidal anti-inflammatory drugs (NSAIDs) have been developed. Such developments have been, and still are, important for pharmacotherapy, but they have also contributed enormously to our current knowledge of physiological and pathological processes. The best example within the field of inflammation is probably the NSAIDs; the availability of a drug such as aspirin made possible the unravelling of the arachidon cascade.

© 1994 Rapid Communications of Oxford Ltd

During the second half of the nineteenth century several scientists predicted that soon insight into structure–activity relationships would allow the design of very selective drugs for virtually all ailments. It was not long before it became clear that these predictions had no value. The simple reason was, of course, that the physiology of the human body is complex, which makes it rather difficult to reach selectivity. In the 1930s the famous pharmacologist Clark sighed that the knowledge on drug activity was still very limited and that the only positive thing was that scientists were aware of their ignorance in this respect.

However, since the turn of the century the ideas about the mechanisms involved in drug actions had changed; the receptor concept was introduced (Langley, Ehrlich). In the 1930s Clark proposed his so-called 'occupancy theory': the effect of a drug is determined by the level of occupation of the receptor by the drug.

It was not until the 1950s and 1960s that really new approaches emerged. In this period pharmacology became a molecular science. In particular, the research group of Ariëns showed the advantages of tests in which direct interaction between a drug and its receptor could be established and which allowed a simple numerical value for biological activity to be obtained. The publication in 1964 of Ariëns' book *Molecular Pharmacology*[1] is to be considered a milestone in the history of drug research.

Another major development concerns the introduction of computers. The use of computers enabled the calculation of quantitative equations in which a given biological activity of individual members of a series of chemically and pharmacologically related substances is related with, for example, parameters that describe the chemical and physical properties of the compounds. The so-called method of multiregression analysis, introduced in 1961 by Hansch,[2] to calculate quantitative structure–activity relationships (QSARs) is as important as the change of pharmacology into pharmacological science.

From the 1960s onwards the search for new medicinal agents started with the concept of a mechanism of action. The search was not simply for an antihypertensive, but for an adrenoceptor blocker; not just for an antiasthmatic, but for an inhibitor of the release of histamine from mast cells. Since then several new types of drugs have been developed, e.g. $\beta_2$-agonists and $\beta_1$-antagonists, $H_2$-blockers, ACE-inhibitors, and proton-pump-blockers.

The trend which developed strongly since then was the need for high selectivity and low toxicity. An extremely nice example of increased selectivity is the development of $\beta_2$-agonists for treating asthma. The old adrenergic agonist ephedrine (from a traditional Chinese preparation) is an effective bronchodilator (via $\beta_2$-receptors, as we know now), but has serious side-effects. It reaches the brain (stimulating potentials for addiction), it is hypertensive ($\alpha_1$) and increases the heart frequency ($\beta_1$). Adrenaline, which was later introduced, has no CNS effects (does not reach the brain), but still has other drawbacks. With isoprenaline (no $\alpha_1$-effect) the effects on the blood pressure were abolished and the subsequent development of salbutamol (no CNS penetration, no $\alpha_1$, no $\beta_1$) as a pure $\beta_2$-agonist led to a compound with still the same main effects as ephedrine, without any of its major side effects, however.

Other examples are the effective and selective antiviral agents (only viricidal in infected cells as the given compound is activated by an enzyme which is introduced by the virus itself), the new selective serotonin agonists (like sumatriptan) or the inhibitors of the $H^+K^+$ATP-ase (omeprazole); many other examples could be given.

It is very difficult to design non-toxic compounds. A major reason is that toxicity is almost always caused by several interactions between the compound (or its metabolites) and the biological system, whereas, in ideal cases, a pharmacological effect is monofactorial. However, the recent development of toxicology into a molecular science (just as happened with pharmacology earlier) will surely contribute to the possibilities of designing better medicines.[3]

Currently, two major new trends are becoming more and more important: the use of the 'big' computers and the application of the techniques of molecular biology. Nowadays, computers are much more than just instruments for complicated calculations. The application of computer graphics to visualize the 3D-structure of receptors (e.g. enzymes, obtained by crystallization) and the interaction of the medicinal agent with the receptor is very attractive for designing better molecules (lead optimalization). These days it is still necessary to have a lead structure but it is expected that it will not take long before it is possible, when the structure of the receptor is available, to have completely new structures generated by the computer (lead generation).

The importance of molecular biology for drug research seems to have been overestimated in the 1980s. Some years ago it was believed that the future of medicinal treatment was in the hands of molecular biology. Now we know that this was a wrong conclusion. A research director of a pharmaceutical company once said during a symposium, 'The application of molecular biology will not lead to really new drugs, as molecular biology concerns processes, not products'. The significance of molecular biology, however, can easily be underestimated as well. The application of molecular biology contributes in several ways to drug research.

- Insight into biological processes, both physiological and pathological

- Elucidation of the structure of receptors; detection of 'new' receptors.
- Production of antibodies (therapy, targeting, vaccines).
- Introduction of gene therapy.

Thanks to the results from molecular biological investigations we know now much more about the structure of receptors, the signal transfer mechanisms, the de- and resensitization events and the so-called cross-talk between different receptor systems.

Taking all things together it seems that perspectives for the development of new drugs have never been as bright as they are now. So, one would think that it should be rather easy to predict a very bright future for those who want to develop new drugs, and possibly also to indicate which type of drugs might be expected.

Unfortunately, both thoughts are not true. To start with the second one, in the 1970s the results of a so-called Delphi investigation predicted which developments were expected to take place in biomedical sciences, especially in pharmacotherapy.[4] Several predictions were correct (a vaccine against measles, better fibrinolytics, no real progress in treating allergies). Other predictions, however, were absolutely wrong (a vaccine against the common cold, improved therapy for multiple sclerosis, a contraceptive for males). However, it is frustrating to see that several major developments were not foreseen (the report even lacks words like 'molecular biology', 'DNA recombinant techniques', 'prostaglandins', '$H_2$-receptor antagonists', 'ACE-blockers', 'proton-pump-blockers', 'AIDS', 'penicillinase inhibitors').

In fact, during a major conference in London in 1978 it was concluded that medicines had no future at all—and that was when products such as cime-tidine, ranitidine, omeprazole and lovastatin were still to be introduced in pharmacotherapy! So it seems very difficult indeed to make predictions in this field.

But it is also not justified to predict a bright future for new drugs in general. Most new drugs, in fact almost all, are developed by pharmaceutical industries. The future of the pharmaceutical industry, however, is not certain internationally. The main reasons for this uncertainty are simple:

- the financial situation deteriorates (reduced consumption of medicines, lowered prices and profit margins);
- the costs of developing a new drug continue to increase (reduced number of 'hits', requirements more severe).

This situation will eventually lead to:

- a reduction in the number of innovating industries (stronger and on a shorter term than expected previously);
- a situation in which each industry will try to present its new product as 'unique' (delay of publishing to avoid the development of early second generation products by competitors, strengthening the monopolistic position);
- a monopolistic position of industries.

In particular, the delay in publishing data on new developments and key structures will be counter-productive for science and for the perspectives of the pharmaceutical industries in the long term. Clearly, these developments have several causes: the costs for health care are considered to be too high, drug prices should be reduced and drug consumption should be diminished. The industries (both the national associations and the individual houses) defend their position. The way the industries do so is not always easy to follow; they never succeeded to explain clearly, for example, why important price differences for the same drug exist in different countries. Regrettably, the action and reaction situation might lead to a monopolistic position of a very limited number of industries indeed. The rapid disappearance of the large group of relatively small innovating industries will be regretted in due time, but at that time it will be too late. It is this last type of industry which has contributed extensively to the development of second generation drugs, drugs which often show important advantages over the first developed compounds in the given class; moreover, such second generation drugs have a role in making the prices competitive.

But, as every cloud has a silver lining, the situation which emerges now could mean that complex molecules such as peptides would have better chances, as such derivatives reach the mark 'unique' relatively easily. As only these unique products will be allowed to have a high price, such products might become worthwhile developing. These situations open good perspectives especially for peptides involved in immunology, allergy, inflammation and related diseases; of course, major problems (e.g. of pharmacokinetic nature) connected with the use of peptides should be solved.

In the longer term, however, pessimism should dominate. The costs involved with the development of any drug are extremely high. Any company can develop a new drug (including the unique ones, with a high price) only if the return on investment is guaranteed; the large amounts of money needed for developing new drugs can only arise from best-selling drugs (sometimes at prices that seem very high for lay people). As only a few companies will be able to develop such best-sellers, the conclusion can only be that the recent attitude of virtually all national governments to reduce drug consumption will eventually lead to a very strongly reduced rate of development of new drugs. This is a pessimistic point-of-view at the very time when scientific potential for new drug development should give high hopes. One can only hope that this opinion is wrong (which is not very likely) or that the national offices of health will realize that their actions will become counter-productive in due course. Maybe it should not be left to the pharmaceutical companies to explain how the real situation is, maybe the independent scientific community should raise its voice a little louder, not to assist the industry, but to contribute to the perspectives of health care.

### References

1. Ariëns EJ (ed.), *Molecular Pharmacology*. New York, London: Academic Press, 1964.
2. Hansch C. *A quantitative approach to biochemical structure-activity relationships*. *Acc Chem Res* 1969; **2**: 232.
3. Marshall E. *Toxicology goes molecular*. *Science* 1993; **259**: 1394–1398.
4. Geneesmiddelen voor de wereld van morgen.. Utrecht: Stichting Voorlichtingscentrum Farmaceutische Industrie, 1990.
